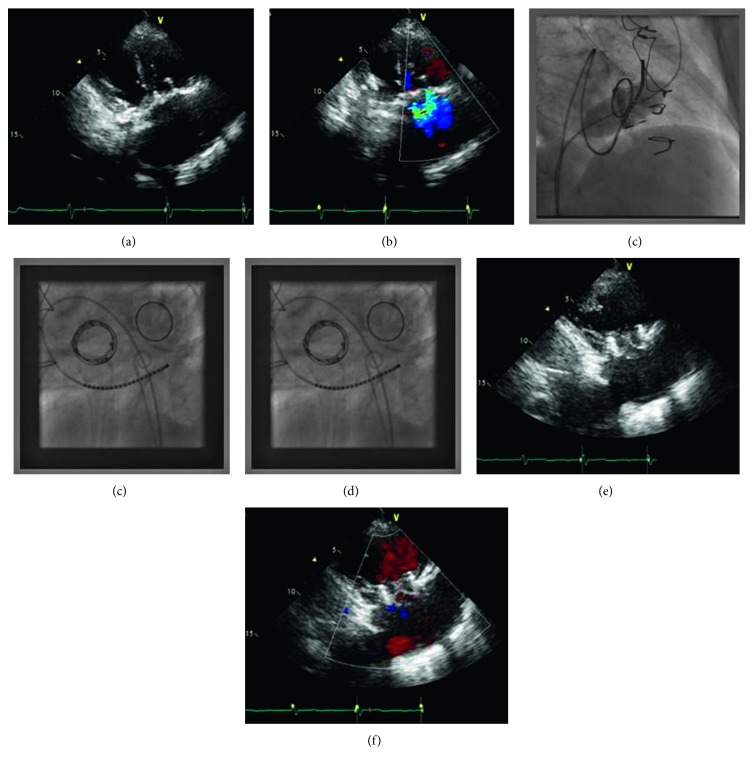# The Use of Adenosine to Enable Safe Implantation of Transcatheter Tricuspid Valve

**DOI:** 10.1155/2017/2760580

**Published:** 2017-11-16

**Authors:** Vicki Zeniou, Shmuel Chen, Mony Shuvy, David Luria, Chaim Lotan, Haim D. Danenberg

**Affiliations:** Hadassah Hebrew University Medical Center, Jerusalem, Israel

## Abstract

High precision is necessary during percutaneous transcatheter heart valve implantation. The precision of the implantation has been established by increasing the heart rate (usually to 200 beats per minute) to the point of significantly reduced cardiac output and thus minimizing valve movement. Routinely, this tachycardia is induced by rapid pacing. Here we report a case of failure to pace during valve-in-valve (VIV) Edwards Sapien XT implantation in the tricuspid valve position. Transient cardiac arrest was induced by intravenous adenosine injection enabling accurate valve implantation.

## 1. Introduction

Transcatheter valve-in-valve (VIV) procedures are relatively new procedures performed increasingly in degenerative bioprosthetic valves as an alternative to surgical replacement. The procedure has challenging technical aspects and requires high level of accuracy. During valve implantation, accuracy is achieved by rapid pacing, which reduces cardiac output and minimizes involuntary valve movement. For this reason, a temporary electrode is placed in the right ventricle.

For tricuspid VIV procedures, pacing may be induced by an electrode placed either in the left ventricle or in the coronary sinus. An attempt may also be made to deploy the valve without pacing due to the low flow velocity in the right ventricle outflow tract.

This is a case of failed rapid pacing during tricuspid VIV implantation. Adenosine was used as an alternative to create temporary heart block, enabling accurate valve deployment during the cardiac arrest period. Adenosine inhibits atrioventricular (AV) node conduction, leading to a short-acting AV block and temporary cardiac asystole [1].

## 2. Case Report

A 63-year-old female with a medical history of rheumatic heart disease, ischemic heart disease, chronic atrial fibrillation, past stroke, and cerebral palsy, who underwent mechanical mitral valve replacement and biological tricuspid valve replacement (Edwards Lifesciences, Perimount 29 mm) at the age of 55, was currently admitted with shortness of breath and severe pitting leg edema. Echocardiography revealed moderate aortic stenosis (mean gradient 30 mmHg), good function of the mechanical mitral valve, severe (free) tricuspid valve regurgitation, and significant tricuspid valve stenosis (Figures 1 and 1). The maximal velocity through the tricuspid valve was measured to be 2.06 m/s with a calculated maximal pressure gradient of 17 mmHg and a mean pressure gradient of 11 mmHg. A right heart catheterization that was performed verified these measurements. STS score for mortality was 9%, and the patient was considered by the heart team to be extremely high risk for surgery. The patient was thus referred for percutaneous transfemoral tricuspid VIV implantation under conscious sedation. Due to normal right-ventricular contraction and high systolic pressures, it was decided to perform the procedure with rapid pacing. Several options for placement of the electrode were considered. Insertion of a right-ventricular electrode is associated with the risk of it being jailed between the old and new prostheses. Furthermore, insertion of a left-ventricular electrode is technically difficult due to the presence of aortic stenosis. In addition, rapid right-atrial pacing did not allow for proper capture of the ventricle. It was decided to place a temporary electrode in the coronary sinus for rapid pacing, but again rapid ventricular pacing could not be achieved due to technical difficulties. Therefore, cardiac arrest was attempted with 6 mg of adenosine; however, this dose was not adequate, and thereafter cardiac arrest was induced with bolus injection of 18 mg of adenosine through a peripheral vein access. Asystole was induced, and a balloon expandable valve (Edwards Lifesciences, Sapien XT 29 mm) was deployed in the tricuspid valve bioprosthesis (Figures 1 and 1). Asystole lasted for nine seconds, allowing slow and accurate balloon inflation and valve deployment. Asystole was followed by slow resumption of cardiac rhythm at a heart rate of 60 bpm. Recovery was immediate, postprocedural course was uneventful, and echocardiography demonstrated normal left- and right-ventricular function, with no significant tricuspid valve stenosis (mean 5 mmHg) and mild tricuspid paravalvular regurgitation (Figures 1 and 1). Echocardiographic follow-up of the patient a year after the procedure showed good global systolic function, with mild paravalvular leak of the tricuspid valve. Heart movement during the cardiac cycle can impact the accuracy of valve implantation, resulting in possible displacement and subsequently increasing the rate of complications.

## 3. Discussion

Cardiac movement might influence the height of implantation in relation to the aortic annulus and increase the probability of paravalvular leak and atrioventricular conduction disturbances, which, in turn, may negatively influence the prognosis of the patient. These movements mainly have an effect on balloon expandable valves, and therefore rapid pacing is considered mandatory during such procedures. Adenosine administered intravenously can induce transient cardiac arrest by inhibiting AV nodal conduction. Having a half-life from 10 to 30 seconds, its effect is achieved rapidly after injection (10–15 seconds) and lasts for several seconds (the drug is cleared from the plasma within 30 seconds). Thus, it has suitable pharmacological features for potential use to induce heart arrest during percutaneous cardiac interventions. The adverse effects are self-limiting, and during the procedure there was no case of bronchospasm.

The literature reveals adenosine being successfully used to enhance accurate deployment of stent grafts in the thoracic aorta [2, 3] and in the implantation of an ostial coronary stent [4]. Davidavicius et al. performed a prospective pilot study and found the use of adenosine safe and feasible to achieve cardiac arrest in 20 patients who underwent TAVI with balloon valvuloplasty in whom rapid pacing would be not well tolerated [5]. Furthermore, it has been reported that adenosine has been used in extracardiac procedures. Pile-Spellman et al. used a rapid injection of 64 mg of adenosine to induce rapid reversible high atrioventricular block and hypotension in order to effectively and safely deposit glue in cerebral arteriovenous malformations during microsurgical resection [6]. After this, Groff et al. published a case in which 6 mg in addition to another 12 mg of adenosine was administered through a central venous catheter to induce hypotension and collapse of the aneurysm in an arteriovenous malformation allowing for closure with a clip [7]. To our knowledge, the current report is the first description of adenosine-induced temporary heart block during percutaneous valve implantation. As it was described above, adenosine could safely enable valve deployment during the tricuspid VIV procedure during which other techniques of reducing cardiac output were unavailable or difficult to pursue.

## Figures and Tables

**Figure 1:**